# Measurement of Quantum Yields of Monolayer TMDs Using Dye-Dispersed PMMA Thin Films

**DOI:** 10.3390/nano10061032

**Published:** 2020-05-28

**Authors:** Shrawan Roy, Anir S. Sharbirin, Yongjun Lee, Won Bin Kim, Tae Soo Kim, Kiwon Cho, Kibum Kang, Hyun Suk Jung, Jeongyong Kim

**Affiliations:** 1 Department of Energy Science, Sungkyunkwan University, Suwon 16419, Korea; shrawanroy4@gmail.com (S.R.); anirsyahmi@gmail.com (A.S.S.); anamess@naver.com (Y.L.); 2 School of Advanced Materials Science & Engineering, Sungkyunkwan University, Suwon 16419, Korea; kwbin7199@naver.com (W.B.K.); hsjung1@skku.edu (H.S.J.); 3 Department of Materials Science and Engineering, Korea Advanced Institute of Science and Technology (KAIST), Daejeon 34141, Korea; taesookim@kaist.ac.kr (T.S.K.); chokw410@kaist.ac.kr (K.C.); kibumkang@kaist.ac.kr (K.K.)

**Keywords:** quantum yield, molybdenum disulfide, molybdenum diselenide, tungsten disulfide, tungsten diselenide, 2D semiconductors, photoluminescence

## Abstract

In general, the quantum yields (QYs) of monolayer transition metal dichalcogenides (1L-TMDs) are low, typically less than 1% in their pristine state, significantly limiting their photonic applications. Many methods have been reported to increase the QYs of 1L-TMDs; however, the technical difficulties involved in the reliable estimation of these QYs have prevented the general assessment of these methods. Herein, we demonstrate the estimation of the QYs of 1L-TMDs using a poly methyl methacrylate (PMMA) thin film embedded with rhodamine 6G (R6G) as a reference specimen for measuring the QYs of 1L-TMDs. The PMMA/R6G composite films with thicknesses of 80 and 300 nm demonstrated spatially homogeneous emissions with the incorporation of well-dispersed R6G molecules, and may, therefore, be used as ideal reference specimens for the QY measurement of 1L-TMDs. Using our reference specimens, for which the QY ranged from 5.4% to 22.2% depending on the film thickness and R6G concentrations, we measured the QYs of the exfoliated or chemical vapor deposition (CVD)-grown 1L-WS_2_, -MoSe_2_, -MoS_2_, and -WSe_2_ TMDs. The convenient procedure proposed in this study for preparing the thin reference films and the simple protocol for the QY estimation of 1L-TMDs may enable accurate comparisons of the absolute QYs between the 1L-TMD samples, thereby enabling the development of a method to improve the QY of 1L-TMDs.

## 1. Introduction

Layered transition metal dichalcogenides (TMDs) with chemical formulas of MX_2_ (wherein M represents a transition metal atom, such as Mo or W, and X represents a chalcogen atom, such as S, Se, or Te) have been extensively investigated for fundamental science and device applications [1,2]. These monolayer transition metal dichalcogenides (1L-TMDs) possess direct band gaps, which make them highly applicable in the field of optoelectronics such as light-emitting diodes [3], phototransistors [4], photo-detectors [5,6], and lasers [7]. However, the photoluminescence (PL) quantum yields (QYs) of pristine 1L-TMDs are typically less than 1%, significantly limiting their application in optoelectronic devices [8]. A high density of structural defects in these systems has been proven to be the main cause of the low QYs of 1L-TMDs [9,10,11,12,13]. Methods to improve the QYs of 1L-TMDs through the treatment of these defects using bis(trifluoromethane) sulfonimide (TFSI) [13,14,15,16], hydrohalic acids (HBr) [17], poly(4-styrenesulfonate) [18], and light illumination [19,20,21] have been reported. However, the effectiveness of these methods is not clear without the general assessment of the QYs of 1L-TMDs. In particular, mere factors indicating PL enhancement do not represent how effective these treatments are because the sample conditions are vastly different. For example, TMD samples with low QYs tend to exhibit higher PL enhancements when the same treatment is used [14]. Therefore, for the accurate comparison of the treatments developed to increase the PL or QY of 1L-TMDs, the reliable and general measurement of the QYs of 1L-TMDs is critically important.

The QY represents the efficiency of the conversion of absorbed photons into emitted photons [22,23], and it has been estimated by directly measuring the absorptions and emissions of the photons using an integrating sphere [24,25,26]. However, such a direct measurement of the absolute QY is not applicable for 1L-TMDs because of their small sizes and significantly low QYs. Alternatively, the QY of the reference sample is first measured using the integrating sphere. The PL intensity and absorption data of the reference sample are then measured using microscopes for comparison with data obtained from the 1L-TMD samples using the same experimental conditions [27,28,29,30]. In this method, the reference samples are usually thin films made of fluorescent organic dyes, such as 3-borylbithiophene derivatives [27,28,29,30] or rhodamine 6G (R6G) [1,13,14]. The thicknesses and emission profiles of the reference films must be spatially uniform at micro-scale dimensions to permit comparison with the local PL of micro-sized 1L-TMDs. R6G in low-concentration solutions has a QY close to 100% [1,31,32]; however, this dye tends to exhibit an extremely low QY in its solid form due to fluorescence quenching that originates from the aggregation of the solid particles [33]. Therefore, they are undesirable for use as reference samples. Previously, 3-borylbithiophene derivatives have been used as reference films [28,29,30]; however, controlling the uniform dispersion of organic molecules in a matrix is technically difficult. Therefore, for the reliable measurement of the QY of 1L-TMDs, the method of preparing thin films containing uniformly dispersed organic dyes is still in high demand.

Herein, we prepared thin reference films with nanoscale thicknesses and the spatially uniform emissions of R6G molecules that are embedded in a poly methyl methacrylate (PMMA) matrix. These films were used for the relative estimation of the QYs of 1L-TMDs, including those of 1L-WS_2_, 1L-MoSe_2_, 1L-MoS_2_, and 1L-WSe_2_. The results were consistent between measurements using two different thicknesses of reference specimens.

## 2. Materials and Methods

### 2.1. Fabrication of Thin Films of R6G Embedded within a PMMA Matrix

First, 0.47906 g of R6G powder was dispersed in a PMMA solution (950 PMMA C4, MicroChem Corp (Westborough, MA, USA)) to prepare a 10^−2^ M concentration of R6G. Here, the PMMA solution was used as a solvent for the R6G molecules. Next, the 10^−2^ M concentration of R6G dispersed in a PMMA solution was diluted to 5 × 10^−5^ M stepwise to obtain various concentrations of this solution. A series of thin films with differing R6G concentrations were prepared on quartz substrates via spin coating at a rate of 3000 rpm for 1 min. In a similar manner, a PMMA solution with a concentration lower than that of 950 PMMA C4 (950 PMMA C2, MicroChem Corp (Westborough, MA, USA)) was used to prepare thinner reference films.

### 2.2. Exfoliation and Growth of 1L-TMDs

All 1L-TMDs were exfoliated on quartz substrates using bulk single crystals (2D Semiconductors Inc. Scottsdale, AZ, USA). 1L-MoS_2_ samples were grown using the chemical vapor deposition (CVD) method on Si/SiO_2_ substrates and transferred onto quartz substrates using the wet transfer method, wherein hydrofluoric acid (HF) was used as the SiO_2_ etchant, according to previously reported methods [13,15,34,35]. The CVD-grown 1L-MoS_2_ samples were chemically treated using TFSI (Sigma-Aldrich, St. Louis, MO, USA) molecules after being transferred onto the quartz substrates and further optical characterizations were conducted [13,14].

### 2.3. Quantum Yield, Confocal PL, Raman, Transmission, and Scanning Electron Microscopy Measurements

The QYs of the thin reference films composed of R6G embedded within a PMMA matrix were determined using a commercial QY spectrometer (Quantaurus-QY, Hamamatsu Photonics, Hamamatsu City, Japan) or a lab-made QY measurement system consisting of an integrating sphere (819C-SL-3.3, Newport, Irvine, CA, USA) and a spectrometer equipped with a cooled charge-coupled device (CCD, Acton, MA, USA). The excitation light source was a 150-W Xenon lamp (Hamamatsu Photonics, Hamamatsu City, Japan) with a selectable wavelength or 514 nm laser line, respectively. For the confocal PL and transmission spectroscopy measurements, a lab-made laser confocal microscope combined with a spectrometer was used [15,34,35,36]. The laser light was focused using a 0.9-NA objective lens, and the scattered light was collected using the same objective lens and guided to a 50-cm-long monochromator equipped with a cooled CCD. The excitation laser was a 514-nm-wavelength argon laser line, which was used for the PL measurements. Scanning electron microscopy (SEM, JEM-2100F, JEOL Corp. Tokyo, Japan) with field voltages of 5–20 KeV was used to obtain images of the cross sections of the thin R6G films embedded within a PMMA matrix, as well as to determine the thicknesses of these films.

## 3. Results and Discussion

**Figure 1:**
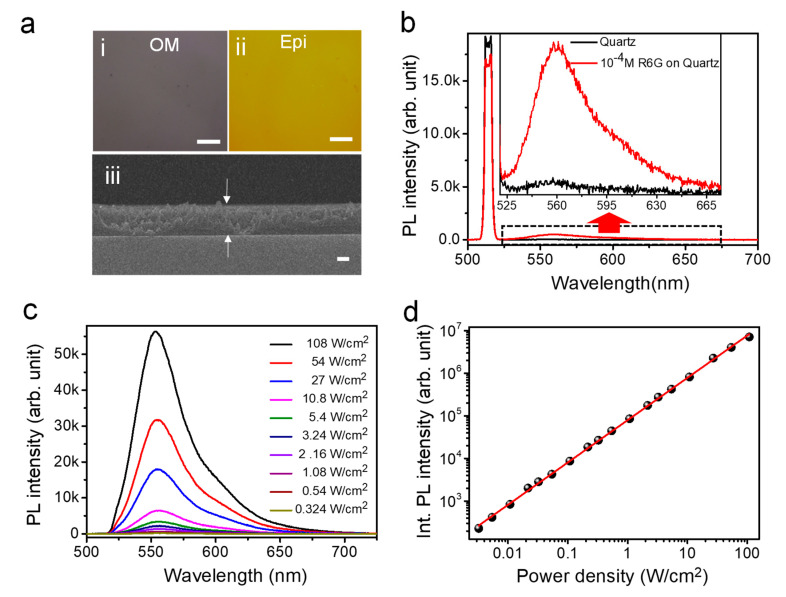
(**a**): (i) Optical microscopy, (ii) epi-fluorescence, and (iii) Scanning electron microscopy (SEM) (cross section showing the uniform thickness of 300 nm) images of a thin film fabricated using a poly methyl methacrylate (PMMA) C4 with 10^−4^ M of rhodamine 6G (R6G) (the scale bars in the optical and SEM images are 5 µm and 100 nm, respectively). (**b**) Photoluminescence (PL) spectrum of a 300-nm-thick film with 10^−4^ M of R6G and a bare quartz substrate measured using an integrating sphere. The inset illustrates a magnified portion of this spectrum for the emission range of R6G. (**c**) Representative confocal PL spectra of the 300 nm thick reference sample with 10^−4^ M of R6G at varied laser intensities under a 514-nm excitation. (**d**) Integrated PL intensity of a 300-nm-thick reference sample with 10^−4^ M of R6G as a function of the laser intensity.

The thicknesses of the thin reference films were controlled using the PMMA concentration in a chlorobenzene solution and the spin coating speed. Two thicknesses of these films, 300 nm (PMMA C4) and 80 nm (PMMA C2), with various concentrations of R6G were fabricated. The optical microscopy (OM), epi-fluorescence (Epi), and SEM (cross section) images of the thin film (300 nm, 10^−4^ M of R6G) exhibit their uniform thickness and fluorescence, as illustrated in Figure 1a(i–iii), respectively. These thin films were loaded into an integrating sphere and excited using a 514-nm wavelength light. The scattered light was collected through an optical fiber connected to the integrating sphere. We found some degradation of PL with high laser intensity over ~11 W/cm^2^, therefore, for every measurement we used new locations of PL collection. An example of the detected spectra obtained with and without the reference sample (for a 300-nm thick thin film with 10^−4^ M of R6G) is depicted in Figure 1b. A reduction in the laser peak corresponds to the absorption ($I_{a}$) by the reference sample, and the peak at ~559 nm (shown in the magnified portion of the spectra in the inset in Figure 1b) represents the emission ($I_{e}$). The QY of the reference sample was estimated using Equation (1):

(1) $$\mathrm{QY}=\frac{I_{e}}{I_{a}}$$

The measured QY of our reference samples range from 5.4% to 22.2% depending on the film thicknesses and R6G concentrations, as presented in Table 1. Considering that the R6G molecules exhibit the highest QY in a solution without aggregation [31,32,33], a similar trend in the thin films composed of R6G embedded in a PMMA matrix may be expected, wherein the QY is higher at lower R6G concentrations. Less QY of thinner reference samples is attributed to the shorter interdistance between R6G molecules and the higher chance of the adsorption of the environmental water or oxygen molecules that could induce the quenching of R6G fluorescence [33,37,38].

The confocal PL spectra of a reference specimen (with a 300-nm thickness and 10^−4^ M of R6G) with varying excitation powers obtained using a 514 nm laser are depicted in Figure 1c. The PL peak at ~553 nm is the characteristic emission peak of R6G, and the linear dependency of the PL intensity on the increasing excitation laser intensity was observed, as depicted in Figure 1d. Similar linear increases of the PL with increasing laser intensities were obtained for the R6G film (80 nm thickness) fabricated using PMMA C2 with 10^−4^ M of R6G (see the Supplementary Materials, Figure S1).

**Figure 2:**
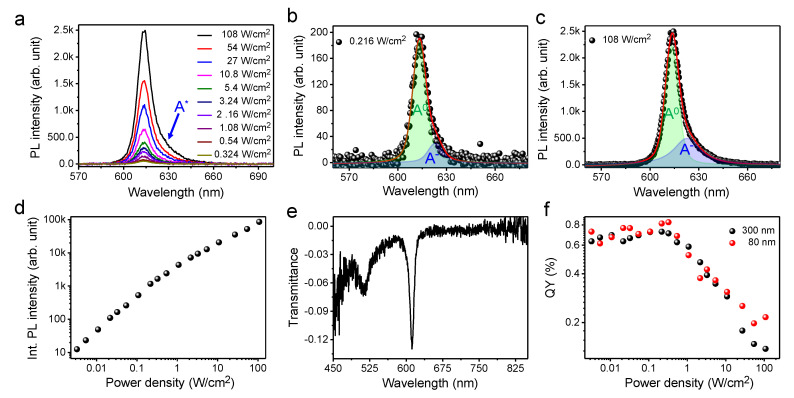
(**a**) Confocal PL spectra of the exfoliated 1L-WS_2_ on a quartz substrate with various laser intensities. Deconvoluted PL spectra of the exfoliated 1L-WS_2_ at (**b**) 0.216 W/cm^2^ and (**c**) 108 W/cm^2^. (**d**) Integrated PL intensity of 1L-WS_2_ as a function of laser intensity. (**e**) Representative transmittance spectrum of 1L-WS_2_. (**f**) Plot of the QYs of 1L-WS_2_ as a function of laser intensity, estimated using 80- and 300-nm-thick reference specimens.

In Figure 2, the QY estimation results for the exfoliated 1L-WS_2_ on a quartz substrate are depicted. The confocal PL spectra obtained with various laser intensities are illustrated in Figure 2a, wherein the PL peak at ~613 nm is consistent with the previous PL results obtained for 1L-WS_2_ [15,39,40] (the Raman spectrum of 1L-WS_2_ that confirm the 1L thickness of this material is provided in the Supplementary Materials, Figure S2a). As shown in Figure 2a, the spectral width of the PL spectra broadens at the shoulder (indicated by A*) as the excitation laser intensity is increased due to increased carrier interactions [15,41]. The deconvoluted PL spectra illustrated in Figure 2b indicate that the spectral weight of the trions is ~15% at 0.216 W/cm^2^, while it increases to ~35% at 108 W/cm^2^ (Figure 2c) due to the increased carrier concentration and subsequent recombination of trions. A nonlinear increase in the PL intensity with increasing laser intensity is observed in the plot depicted in Figure 2d. We estimated the QY of 1L-WS_2_ at low excitation intensity of linear dependence, where the exciton–exciton interactions were negligible [28]. The representative transmittance spectrum of 1L-WS_2_ used to measure the absorption on the quartz substrate is illustrated in Figure 2e, wherein the sharp exciton peak at ~612 nm corresponds to the A exciton of 1L-WS_2_. This is consistent with the obtained PL spectrum and previous results concerning the transmittance of 1L-WS_2_ [42,43]. The PL QY of 1L-WS_2_ was estimated using the following, Equation (2) [28,29,44]:

(2) $$\Phi_{TMD}=\Phi_{Ref}\;\frac{I_{TMD}^{PL}/A_{TMD}}{I_{Ref}^{PL}/A_{Ref}}\;F_{sp}$$

Here,

- Φ*_TMD_* = QY of the TMD to be measured
- Φ*_Ref_* = QY of the reference sample measured with the integrating sphere
- $I_{TMD}^{PL}$ = PL intensity of the TMD measured under the microscope
- $I_{Ref}^{PL}$ = PL intensity of the reference sample measured under the microscope
- *A_TMD_* = Absorption of the TMD measured under the microscope
- *A_Ref_* = Absorption of the reference sample measured under the microscope
- $F_{sp}$ = Calibration factor for the wavelength dependence on the sensitivities of our microscope and spectrometer with respect to the peak emission wavelength of the reference sample (553 nm). Regarding the emission of each 1L-TMDs, *F_sp_* was estimated from the calibration factor vs. wavelength curve shown in Figure S3 in Supplementary Materials. This curve was obtained using 532 nm, 633 nm, and 785 nm lasers in our microscope equipped with a spectrometer, under the same experimental conditions. For our system, this was measured to be 1.17, 1.30, 1.80, and 2.0 for the peak emission at 613 nm of 1L-WS_2_, 658 nm of 1L-MoS_2_, 745 nm of 1L-WSe_2_, and 785 nm of 1L-MoSe_2_, respectively.

The QYs of 1L-WS_2_, estimated using Equation (2), were plotted as a function of the excitation laser intensity in Figure 2f. Here, the QYs were constant until an excitation laser intensity of 0.1 W/cm^2^ was reached because the emissions were solely dominated by the single-body process (exciton recombination) at low excitation powers. The QYs then decreased with increasing excitation intensities due to the activation of many-body processes, such as trion formation and exciton–exciton annihilation [16,45,46]. Noticeably, the estimated QYs of 1L-WS_2_ using both reference samples of different thicknesses (80 and 300 nm) were similar, thereby providing clear evidence of the reliability of using R6G molecules embedded within a PMMA film as standard reference samples for the estimation of the QY of 1L-TMDs. The estimated QY of 1L-WS_2_ is 0.73 ± 0.11%, which is in a similar range to the previously reported QY of 1L-WS_2_ [29].

**Figure 3:**
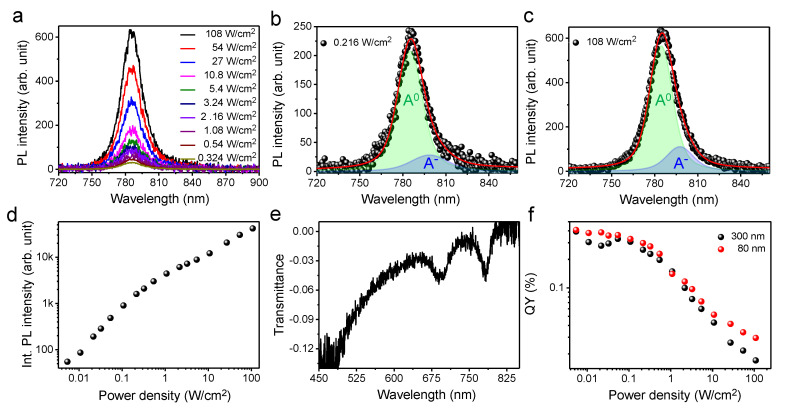
(**a**) Confocal PL spectra of the exfoliated 1L-MoSe_2_ on a quartz substrate with various laser intensities. Deconvoluted PL spectra of the exfoliated 1L-MoSe_2_ at (**b**) 0.216 W/cm^2^ and (**c**) 108 W/cm^2^. (**d**) Integrated PL intensity of 1L-MoSe_2_ as a function of laser intensity. (**e**) Representative transmittance spectrum of 1L-MoSe_2_. (**f**) Plot of the QYs of 1L-MoSe_2_ as a function of laser intensity, estimated using 80- and 300-nm-thick reference specimens.

Figure 3a illustrates the confocal PL spectra of the 1L-MoSe_2_ exfoliated on a quartz substrate with varying laser intensities, wherein the peak at ~785 nm is consistent with previous PL results for 1L-MoSe_2_ (for the Raman spectra, see the Supplementary Materials, Figure S2b) [34,47]. As laser-intensity-dependent PL spectra are illustrated in Figure 3a, the PL spectra are shown to be quite symmetrical even at higher laser intensities in comparison with 1L-WS_2_; however, the influence of the recombination of trions [34] can be clearly observed in the deconvoluted PL spectra illustrated in Figure 3b,c. In these figures, it can be seen that the spectral weight of trion increases from ~16% to ~20% as the laser intensity is increased. Figure 3d depicts the linear dependency of the PL intensity of 1L-MoSe_2_ at low laser intensities, while this behavior becomes nonlinear at high laser intensities [29]. The consistency of the two peaks at ~783 nm and ~694 nm corresponding to the A and B excitons, respectively, in the transmittance spectra of 1L-MoSe_2_ demonstrate the 1L thickness of the MoSe_2_ sample used in this study [48]. The QY of 1L-MoSe_2_ was also estimated using both reference samples (with 80-and 300-nm thicknesses), and the results were plotted against the laser intensity, as depicted in Figure 3f. It can be clearly observed that the QYs estimated using the two reference samples with different thicknesses (80 and 300 nm) are similar. The estimated QY of 1L-MoSe_2_ at low excitation intensity was found to be 0.38 ± 0.02%.

**Figure 4:**
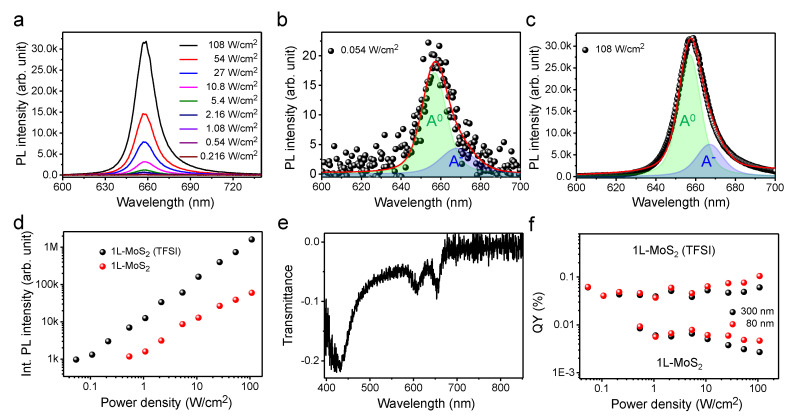
(**a**) Confocal PL spectra of the bis(trifluoromethane) sulfonimide (TFSI)-treated chemical vapor deposition (CVD)-grown 1L-MoS_2_ on a quartz substrate with various laser intensities. Deconvoluted PL spectra of the TFSI-treated CVD-grown 1L-MoS_2_ at (**b**) 0.054 W/cm^2^ and (**c**) 108 W/cm^2^. (**d**) Integrated PL intensities of the TFSI-treated and pristine CVD-grown 1L-MoS_2_ samples as a function of laser intensity. (**e**) Representative transmittance spectrum of the TFSI-treated CVD-grown 1L-MoS_2_. (**f**) Plot of the QYs of the pristine and TFSI-treated 1L-MoS_2_ samples as a function of the laser intensity, which were estimated using 80- and 300-nm-thick reference specimens.

For 1L-MoS_2_, the effect of chemical treatment using TFSI to increase the QY was investigated because TFSI is known to enhance the QY of 1L-TMDs via the repair of the sulfur vacancies [13,14]. The laser-intensity-dependent PL spectra of the TFSI-treated CVD-grown 1L-MoS_2_ on a quartz substrate are depicted in Figure 4a (for the Raman spectra, see the Supplementary Materials, Figure S2c). The peak shown at ~658 nm is consistent with previous PL results obtained for 1L-MoS_2_ [13]. The representative deconvoluted PL spectra at low (0.054 W/cm^2^) and high (108 W/cm^2^) laser intensities are illustrated in Figure 4b,c, respectively. In these figures, it is shown that contribution of the trion recombination is ~25% for the overall emissions at all power ranges measured in this study. The PL spectra of the pristine, CVD-grown 1L-MoS_2_ are depicted in Figure S4 in Supplementary Materials. The PL intensities of the pristine and TFSI-treated 1L-MoS_2_ are depicted in Figure 4d, and the transmittance spectrum of the TFSI-treated 1L-MoS_2_ is illustrated in Figure 4e. In this figure, the peaks at ~655 and ~607 nm correspond to the A and B excitons of 1L-MoS_2_, respectively. The QYs of the pristine and TFSI-treated CVD-grown 1L-MoS_2_ obtained using Equation (2) with both reference samples (of 80 and 300 nm thicknesses) are plotted in Figure 4f. The QYs for the pristine and TFSI-treated samples are estimated to be 0.0093 ± 0.001% and 0.062 ± 0.01%, respectively, indicating the enhancement of QY by TFSI treatment.

**Figure 5:**
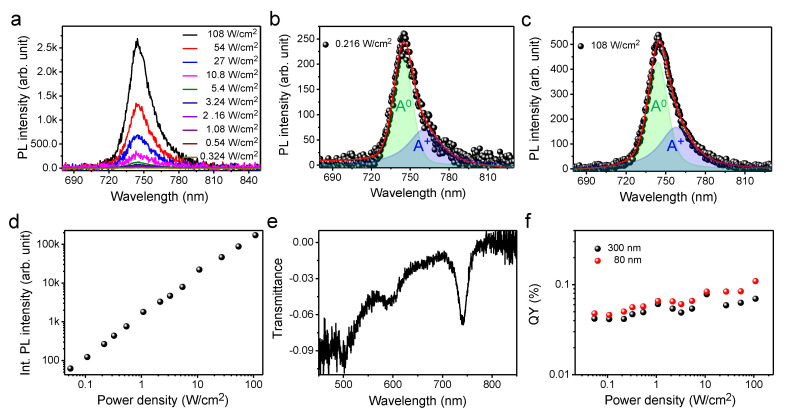
(**a**) Confocal PL spectra of the exfoliated 1L-WSe_2_ on a quartz substrate with various laser intensities. Deconvoluted PL spectra of the exfoliated 1L-WSe_2_ at (**b**) 0.216 W/cm^2^ and (**c**) 108 W/cm^2^. (**d**) Integrated PL intensity of 1L-WSe_2_ as a function of laser intensity. (**e**) Representative transmittance spectra of 1L-WSe_2_. (**f**) Plot of the QYs of 1L-WSe_2_ as a function of laser intensity, estimated using 80- and 300-nm-thick reference specimens.

The QY estimation results of the exfoliated 1L-WSe_2_ are shown in Figure 5. The PL spectra of 1L-WSe_2_ exfoliated on a quartz substrate with varying laser intensities are depicted in Figure 5a (for the Raman spectra, see the Supplementary Materials, Figure S2d). In this figure, the peak at ~745 nm is consistent with the previous PL results obtained for 1L-WSe_2_ [29,34,35]. The shapes of the PL peaks and deconvoluted spectra illustrated in Figure 5b,c indicate that the contributions of the trion recombination in the overall emissions is ~42% for all power ranges measured in this study. The PL intensity is also shown to be linearly dependent on the increasing laser intensity, as depicted in Figure 5d. The transmittance spectrum of 1L-WSe_2_ illustrated in Figure 5e exhibits a sharp peak at ~742 nm, which corresponds to the A exciton and is in good agreement with previous results obtained for the transmittance of 1L-WSe_2_ [48]. The QY of 1L-WSe_2_ was estimated using both reference samples (with 80- and 300-nm thicknesses) and plotted against the laser intensity, as depicted in Figure 5f. The QY values obtained using both reference samples were similar, which is consistent with the results obtained for 1L-WS_2_, 1L-MoSe_2_, and 1L-MoS_2_. The QY of 1L-WSe_2_ was estimated to be 0.066 ± 0.02%. The estimated QYs of the CVD-grown 1L-MoS_2_ and 1L-WSe_2_ were less than the previously reported values for these materials. This may be attributable to the quality of the samples used in this study because the QY of 1L-TMDs mainly depends on the crystal quality of the samples [13,14].

## 4. Conclusions

We fabricated R6G-embedded PMMA thin films with two different thicknesses (80 and 300 nm) as reference samples that were used for the relative estimation of the PL QY of 1L-TMDs. The estimated QY results of 1L-WS_2_, 1L-MoSe_2_, 1L-MoS_2_, and 1L-WSe_2_ were highly consistent between the measurements obtained using the reference specimens with two different thicknesses and assert the reliability for the estimation of QY using these reference samples and the applicability for other micro-sized nanomaterials.

## Figures and Tables

**Table 1:** The photoluminescence (PL) quantum yields (QYs) of the thin reference films measured using integrating sphere for varied concentrations of rhodamine 6G (R6G) and two different thicknesses.

Concentration (M)	Thickness	10^−2^	5 × 10^−3^	10^−3^	5 × 10^−4^	10^−4^	5 × 10^−5^
Quantum Yield (%)	300 nm	14.5	17.8	19.5	20.3	20.6	22.2
	80 nm	5.4	6.4	6.2	9.1	9.5	10.8

## Supplementary Materials

The following are available online at https://www.mdpi.com/2079-4991/10/6/1032/s1, Figure S1: Laser-intensity-dependent PL spectra with optical microscopy, epi fluorescence and SEM images of 80-nm-thick R6G-embedded PMMA thin film with 10^−4^ M, Figure S2: Raman spectra of 1L-WS_2_, 1-MoSe_2_, 1L-MoS_2_ and 1L-WSe_2_., Figure S3: Plot of calibration factor vs wavelength; Figure S4: Laser-intensity-dependent PL spectra and transmittance spectra of pristine CVD grown 1L-MoS_2_.
