# Supplementary material for: Measurement of Quantum Yields of Monolayer TMDs Using Dye-Dispersed PMMA Thin Films
Source: Nanomaterials (Basel). 2020 May 28;10(6):1032. doi: 10.3390/nano10061032 (PMC7353022; doi:10.3390/nano10061032)
Supplement: Supplementary file 1 [file nanomaterials-10-01032-s001.pdf]

Supplementary materials

Measurement of Quantum Yields of Monolayer TMDs Using Dye-Dispersed PMMA Thin Films

Shrawan Roy [1], Anir S. Sharbirin [1], Yongjun Lee [1], Won Bin Kim [2], Tae Soo Kim [3], Kiwon Cho [3], Kibum Kang [3], Hyun Suk Jung [2] and Jeongyong Kim [1,*]

[1] Department of Energy Science, Sungkyunkwan University, Suwon 16419, Korea; shrawanroy4@gmail.com (S.R.); anirsyahmi@gmail.com (A.S.S.); anamess@naver.com (Y.L.)

[2] School of Advanced Materials Science & Engineering, Sungkyunkwan University, Suwon 16419, Korea; kwbin7199@naver.com (W.B.K.); hsjung1@skku.edu (H.S.J.)

[3] Department of Materials Science and Engineering, Korea Advanced Institute of Science and Technology (KAIST), Daejeon 34141, Korea; taesookim@kaist.ac.kr (T.S.K.); chokw410@kaist.ac.kr (K.C.); kibumkang@kaist.ac.kr (K.K.)

* Correspondence: j.kim@skku.edu

Received: 17 April 2020; Accepted: 25 May 2020; Published: date

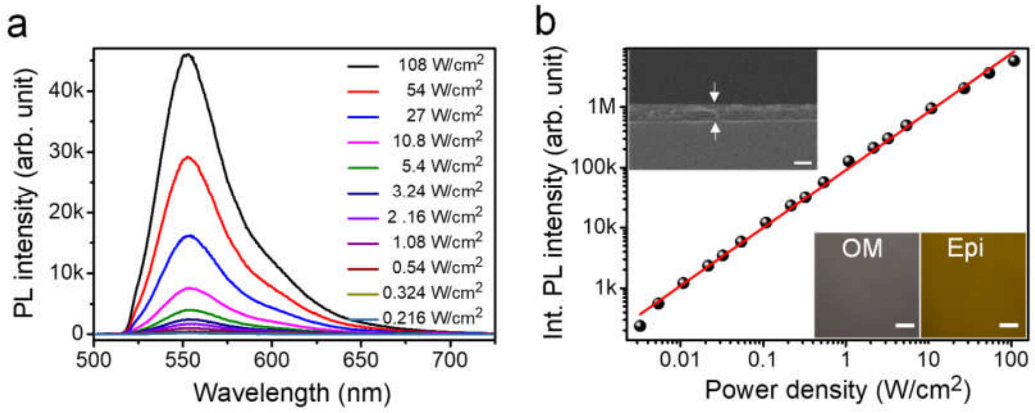

Figure S1. (a) Representative confocal PL spectra of the 80-nm thick reference sample with 10^{-4} M of R6G at various laser intensities under 514-nm excitation. (b) Integrated PL intensity of the 80-nm thick reference sample with 10^{-4} M of R6G as a function of laser intensity. The insets depict the optical microscopy, epi-fluorescence (lower), and SEM cross-sectional (upper) images of the 80-nm thick reference sample with 10^{-4} M of R6G. The scale bars in the optical and SEM images are 5 μm and 100 nm, respectively.

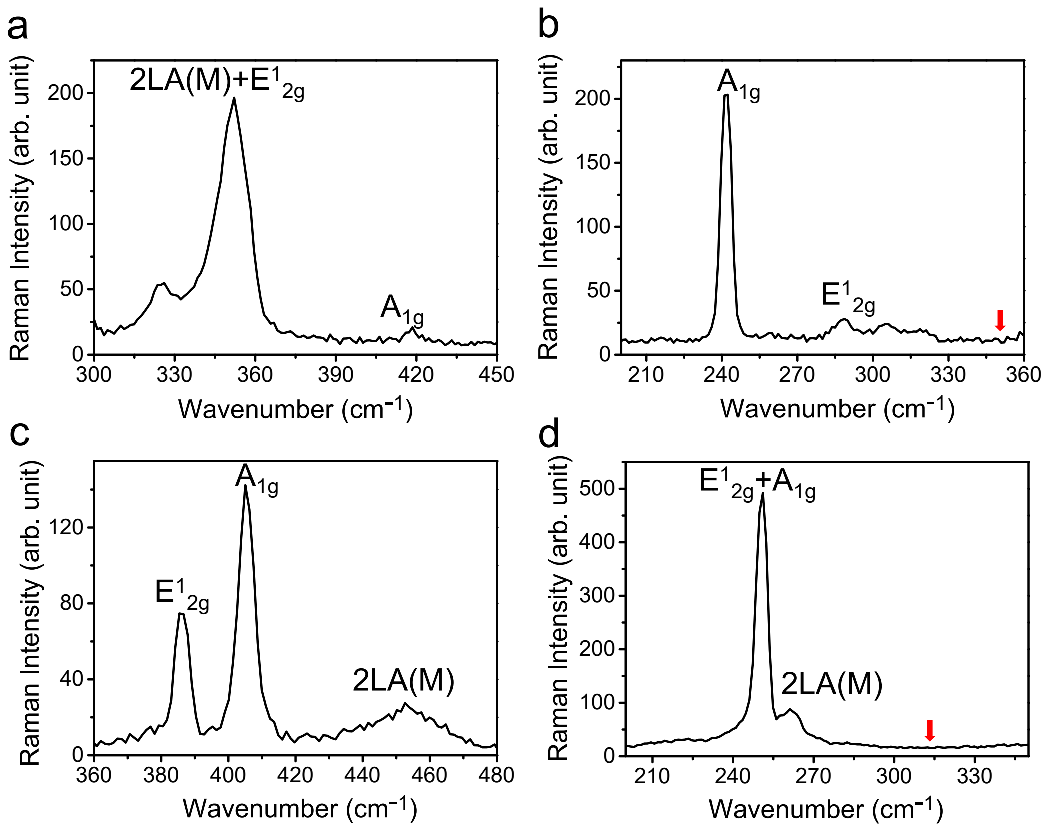

Figure S2. Raman spectra of (a) 1L-WS_2, (b) 1L-$MoSe_2$, (c) 1L-MoS_2, and (d) 1L-WSe_2 on quartz substrates. The peak positions of 2LA(M)+E^1_{2g} and A_{1g} are 352 cm^{-1} and 418 cm^{-1}, respectively in (a); the peak position difference between E^1_{2g} and A_{1g} is 19 cm^{-1} in (c); and the absence of the B_{2g} mode indicated by the red arrows in (b) and (d) show that the TMD samples used in this study are monolayers.

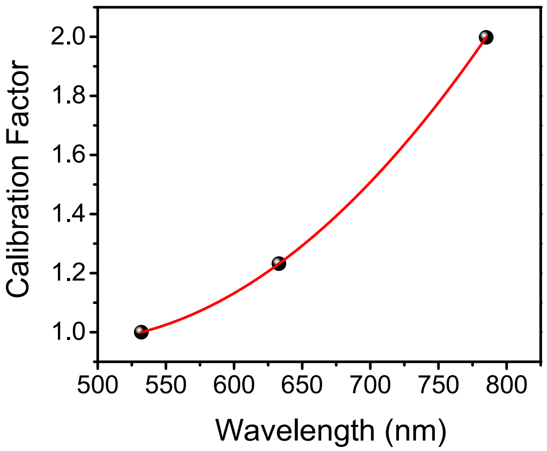

Figure S3. Calibration factor vs wavelength curve obtained using 532 nm, 633 nm and 785 nm diode lasers in this study.

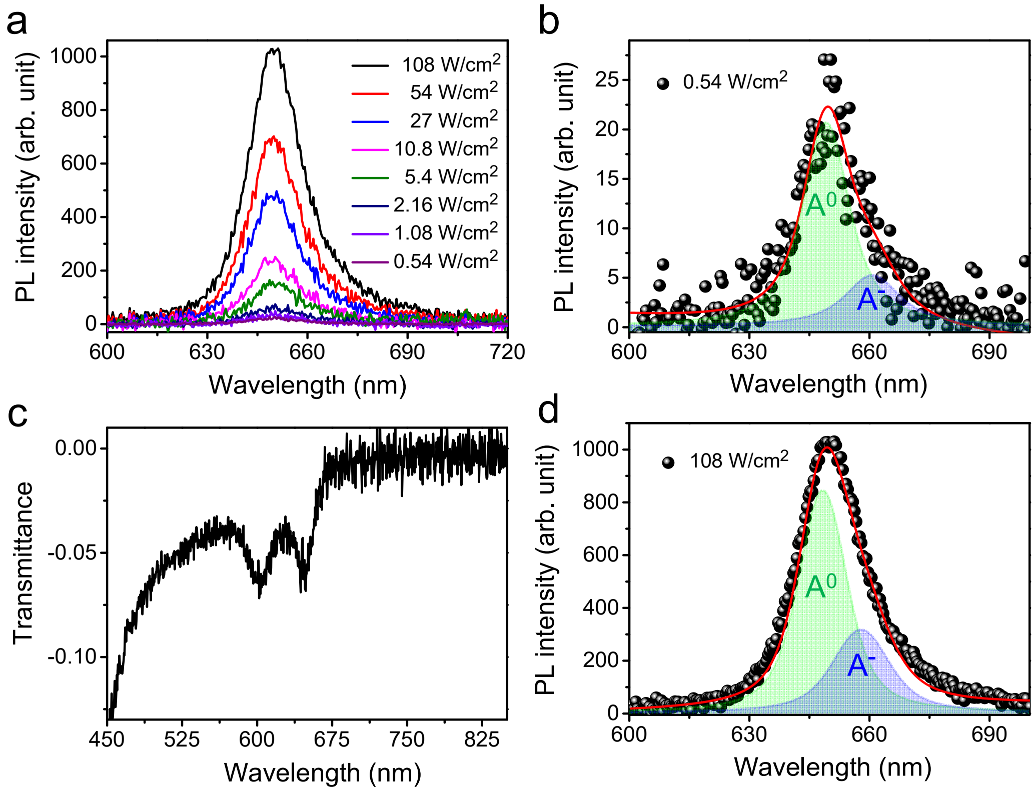

Figure S4. (a) Confocal PL spectra of the CVD-grown 1L-MoS_2 on a quartz substrate with various laser intensities. Deconvoluted PL spectra of the CVD-grown 1L-MoS_2 at (b) 0.54 W/cm^2 (A^0 = 77%, A^- = 23%) and (d) 108 W/cm^2 (A^0 = 70%, A^- = 30%). (c) Representative transmittance spectrum of CVD-grown 1L-MoS_2.
